# The Inhomogeneous Characteristics of Evaporation Ducts in the Northern South China Sea Based on Information Entropy

**DOI:** 10.3390/e28040368

**Published:** 2026-03-25

**Authors:** Ning Yang, Debin Su, Yuduo Feng, Tao Wang

**Affiliations:** 1 College of Electronic Engineering, Chengdu University of Information Technology, Chengdu 610225, China; 2 Key Laboratory of Atmospheric Sounding, China Meteorological Administration, Chengdu 610225, China; 3 School of Electronics and Communication Engineering, Sun Yat-sen University, Shenzhen 518107, China

**Keywords:** evaporation duct, inhomogeneous, electromagnetic propagation, information entropy

## Abstract

The inhomogeneity of the evaporation duct significantly influences electromagnetic propagation. Based on observation data from four buoy stations in the northern South China Sea (SCS) and European Centre for Medium-Range Weather Forecasts (ECMWF) data, the Naval Postgraduate School (NPS) model is employed to calculate the evaporation duct height (EDH). The concept of information entropy is used to assess the horizontal inhomogeneity of the evaporation duct and the evaporation duct height entropy (EDHE) is defined as the assessment index. The research findings are as follows: (1) The probability of EDH differences based on statistical methods between stations falling within the range of −2 m to 2 m remains above 60%, with uniformity characteristics showing minimal variation throughout the day. (2) The EDHE can better quantify the horizontal inhomogeneous characteristics of EDH between buoy stations compared to statistical methods. (3) The monthly variation characteristics of EDHE between buoy stations based on ECMWF reanalysis data are quite consistent with actual observations, but it overestimates the EDHE values. Therefore, the EDH derived from ECMWF data leads to an overestimation of inhomogeneity characteristics compared to buoy observations.

## 1. Introduction

Atmospheric ducting is a distinct atmospheric layer structure that leads to abnormal electromagnetic propagation over the sea [1]. The evaporation duct, a unique type of surface duct, is a nearly constant presence above the sea [2]. This duct gives rise to the swift reduction in water content in the near-surface layer as height increases, caused by seawater evaporation [3,4]. Typically occurring at heights below 40 m, it greatly affects the functionality of electromagnetic equipment at sea [5].

When electromagnetic waves become trapped in the atmospheric duct layer, they can propagate along the horizon with lower path loss, enabling beyond-line-of-sight (BLoS) propagation [6,7]. This phenomenon may cause significant errors in radar ranging, angle measurement, and speed measurement, increase radar clutter, and create substantial radar detection blind zones [8]. However, harnessing the trapping properties of electromagnetic waves can facilitate BLoS detection and communication in the shortwave and microwave frequency bands [9,10,11]. For instance, on 13 April 2022, the Russian warship “Moskva” was struck and sunk by two Ukrainian R-360 Neptune anti-ship missiles launched in the Black Sea. According to relevant studies, under the weather conditions prevailing at the time of the missile launch, ground radar managed to detect targets well beyond the typical radar detection range due to anomalous propagation conditions [12]. Hence, understanding the distribution characteristics of atmospheric ducts is vital for numerous naval operations and civilian applications.

Due to its unique climate and surface characteristics, the ocean poses significant challenges for establishing communication base stations. Presently, artificial satellites serve as the primary means of relaying radio waves, facilitating communication among multiple ground stations [13,14]. Nevertheless, satellite communication comes with high costs, susceptibility to weather disruptions, vulnerability to cyber threats, and privacy breaches. Furthermore, environmental concerns such as space debris pose challenges to the sustainability of satellite communication technologies [15]. One possible key to solving this problem is the application of a distributed buoy network [10,16,17].

Since evaporation ducts are nearly permanent in maritime and coastal environments, ducting layer communication is a promising method for BLoS communications especially in naval communications [11]. However, ducting layer studies mainly focus on refractivity estimation techniques and radar path loss calculations [7,8,18,19]. The utilization of the atmospheric ducts as a communication medium has increased in recent years [9,10,20,21,22]. Ergin Dinc and Ozgur B. Akan [11] overview the characteristics and the channel modeling approaches for ducting layer communications, and review the possible utilization of the ducting layer in network-centric operations to empower decision-making for the BLoS operations. Ma et al. [9] have demonstrated that a BLoS microwave link using evaporation ducts in the frequency range of 8 GHz∼12 GHz is a promising alternative scheme for achieving long-range BLoS naval communication without relays by theoretical and experimental explorations. Yang et al. [23] pointed out that communication with evaporation ducts can provide a wide-bandwidth, high-speed, and long-distance propagation method for maritime applications in the South China Sea (SCS). Cruz et al. [24] created a tool to simulate the effects of evaporation ducts on wireless communication networks.

The evaporation duct height (EDH) is a major parameter in microwave propagation prediction models [25]. Observations of evaporation ducts at a single location are commonly used to evaluate radar propagation conditions and are then applied to a large area. This approach may be sufficient for achieving an acceptable level of accuracy over the open ocean, with the assumption of EDH spatial homogeneity [26]. However, the evaporation duct exhibits significant spatial heterogeneity of EDH, which has a significant influence on electromagnetic propagation [27,28,29]. Shi et al. [27] showed that the path loss is significantly higher than that in the homogeneous case when the EDH at the receiver is lower than that at the transmitter, and the horizontal inhomogeneity of the evaporation duct has a significant influence when the EDH is low or when the electromagnetic wave frequency is lower than 13 GHz based on numerical simulation and experimental observation data. Moreover, in communication systems where the transmitter and receiver share a common antenna, there is a phenomenon known as unidirectional data transmission, wherein only one party can receive the signal when the distance between them exceeds a certain threshold [30]. So, when considering BLoS communications in an evaporation duct environment, it is crucial to consider the horizontal inhomogeneity of EDH.

The evaporation duct studies mainly focus on mechanism analysis [4,31,32,33] and inversion [34,35,36]. However, there are few studies that focus on the horizontal inhomogeneity of the evaporation duct. In our previous research [37], we investigated the inhomogeneity characteristics of evaporation ducts in the SCS region using statistical methods, which can reveal the probability distribution of the difference in EDH between two points but are insufficient indicators for assessing the overall inhomogeneity of EDH between different stations. To this end, this paper analyzes the differences in EDH between the four buoys in the northern SCS, and the concept of information entropy is applied to analyze the horizontal heterogeneity of EDH levels using the European Centre for Medium-Range Weather Forecasts (ECMWF) dataset, defining evaporation duct height entropy (EDHE), and exploring the horizontal heterogeneity characteristics of EDH in the northern SCS.

The rest of this paper is structured as follows. Section 2 describes the data from buoy stations and reanalysis data, the evaporation duct model, and EDHE. Section 3 analyzes the horizontal inhomogeneity of the evaporation duct in the northern SCS region, determining the main influencing factors causing the inhomogeneous characteristics of EDH, and Section 4 discusses the relevant results. Finally, the derived conclusions are presented in Section 5.

## 2. Data and Method

### 2.1. Data

#### 2.1.1. Bulk Measurements from Buoy Stations

**Figure 1:**
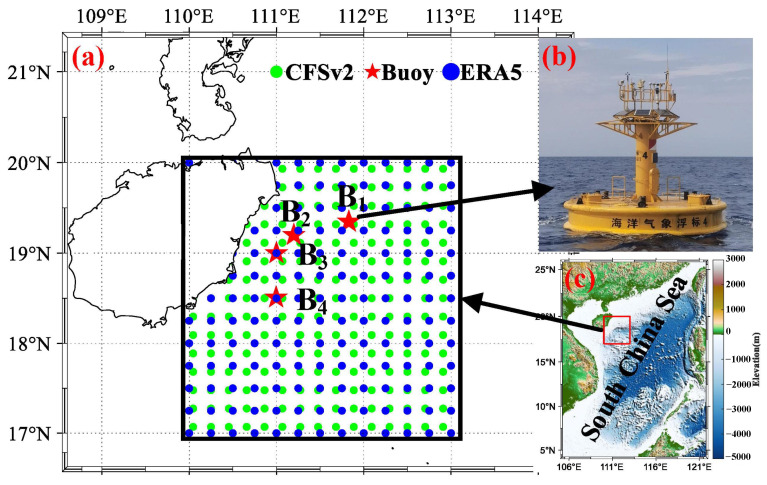
Research area in this paper. (**a**) Location of buoy stations (red Pentagram) and grid points of reanalysis data (the blue dots represent the grid points of ECMWF data, and the green dots represent the grid points of CFSv2). (**b**) Six-meter buoy stations. (**c**) Map of the South China Sea (SCS).

The China Meteorological Administration has established several buoy observation points in the eastern part of Hainan Island to improve the observation of atmospheric and oceanic elements. Among these points, four observation locations (indicated by red pentagrams in Figure 1) fulfill the requirements for calculating the evaporation duct. These four buoy stations are named B_1_, B_2_, B_3_, and B_4_ from north to south, with B_3_ being the closest to the coastline (approximately 60 km away). All four buoys have a height of 6 m. The observation heights for air temperature (AT) and relative humidity (RH) are set to 5 m, wind speed (WS) to 6 m, and air pressure to 2.9 m. Sea surface temperature (SST) is determined using a conductivity, temperature, and depth (CTD) sensors, with its observation height set to 0 m. Detailed information on the sensors for each element can be found in Table 1.

**Figure 2:**
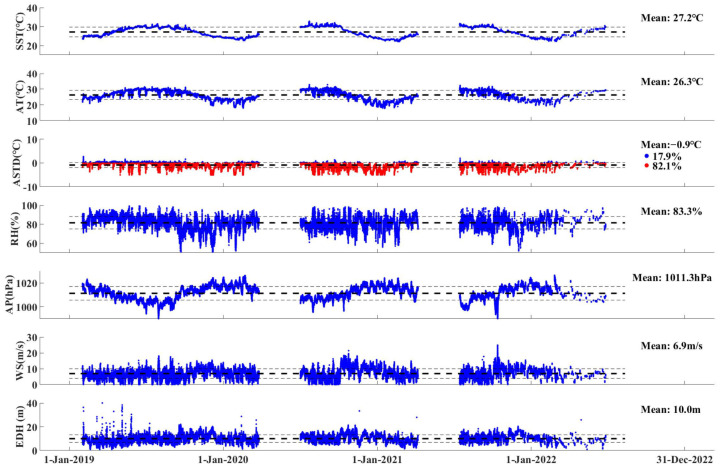
Time series of the B_1_ measurements in the order of SST, AT, ASTD, RH, AP, WS, and EDH from top to bottom. The bold black dotted line and thin dotted line correspond to each variable’s mean value and standard deviation, respectively. The percentage of stable and neutral conditions accounts for 17.9% (blue dots in ASTD) and that of unstable conditions accounts for 82.1% (red dots in ASTD).

To remove singular observations and smooth variability, variable values with a 10 min average measured from January 2019 to December 2022 were used in this paper. Due to the influence of waves, the violent swinging of buoys has a certain impact on SST observations, leading to abnormal SST observations (sudden increase or decrease), which are directly removed. Moreover, there are periods with missing data for various stations and elements because of equipment maintenance (Figure 2).

The time series of the B_1_ measurements in the order of SST, AT, air–sea temperature difference (ASTD), RH, AP, WS, and EDH from top to bottom are shown in Figure 2. Among them, the EDH is calculated using the Naval Postgraduate School (NPS) model (Section 2.2). The mean value (bold dotted line) and the standard deviation (thin dotted line) of each variable are shown in Table 2. The variation trend of the SST matches well with that of the AT. The mean value of the SST is 0.9 °C higher than that of the AT, but the standard deviation of AT is 0.4 °C higher than that of the SST, and the percentage of ASTD < 0 accounts for 82.1%. So, the mean value of the ASTD is −0.9 °C and the standard deviation is 1.0 °C. Furthermore, the mean value and standard deviation of the EDH are 10.0 m and 3.2 m, respectively.

#### 2.1.2. Reanalysis Data

Reanalysis is a systematic approach to producing datasets for climate monitoring and research. It provides the most complete picture currently possible of past weather and climate. This includes a blend of observations (including but not limited to radiosonde, satellite, buoy, aircraft, and ship reports) with past short-range weather forecasts rerun with modern weather forecasting models to produce a new best estimate of the state of the atmosphere.

ERA5 is a fifth-generation ECMWF reanalysis of the global climate and weather for the past 8 decades, with a spatial resolution of 0.25° × 0.25°. Data are available from 1940 onwards, and it replaces the ERA-Interim reanalysis [38]. The National Centers for Environmental Prediction (NCEP) Climate Forecast System (CFS) is initialized four times per day (0000, 0600, 1200, and 1800 UTC), with a spatial resolution of 0.2° × 0.2°. The NCEP upgraded the CFS to version 2 (CFSv2) on 30 March 2011. This is the same model that was used to create the NCEP Climate Forecast System Reanalysis (CFSR) [39]. Specific information on the reanalysis data used in this paper is shown in Table 3, and the grid points of the data are shown in Figure 1a, wherein the blue dots represent the grid points of ERA5 data, and the green dots represent the grid points of CFSv2. Moreover, the distance weighting method is employed to calculate these elements to obtain the AT, AP, RH, and SST of reanalysis data at buoy stations.

### 2.2. Evaporation Duct Model

Due to the challenges of achieving regionalization and high-spatiotemporal-resolution detection of EDH at sea, current approaches mainly involve measuring meteorological parameters near the sea surface and sea surface temperature, or utilizing reanalysis data and predicting based on the evaporation duct model. Evaporation duct models are all based on the Monin–Obukhov Similarity Theory (MOST) or Liu–Katsaros–Businger (LKB) theory [40], such as the Paulus–Jeske (PJ) [41], Babin–Young–Carton (BYC) [25], Naval Research Laboratory (NRL) [42], NPS [41], and Russian State Hydrometeorological University (RSHMU) [43] models. However, the NPS and PJ models are mainly used to calculate EDH. Among these, the NPS model is widely applied for its superior accuracy in evaporation duct and electromagnetic propagation research.

The NPS model effectively characterizes the refractive index profile of evaporation ducts over the sea. By computing AT, specific humidity, and AP profiles, the model leverages atmospheric refractivity and its correlations to determine the refractive index profile of the evaporation duct and ascertain its height. The detailed information and mathematical expressions of AT, specific humidity, and water vapor pressure as functions of height *z* are available in reference [41]. To enhance the NPS model’s performance in neutral and atmospherically stable conditions, this study incorporates the stability function proposed by Grachev et al. [44]:

(1) $$\begin{array}{c} \psi_{h}=-\frac{b_{h}}{2}\ln\left(1+c_{h}\xi +\xi^{2}\right)(-\frac{a_{h}}{B_{h}}+\frac{b_{h}c_{h}}{2B_{h}})\\ \times (\ln\frac{2\xi +c_{h}B_{h}}{2\xi +c_{h}+B_{h}}-\ln\frac{c_{h}-B_{h}}{c_{h}+B_{h}}) \end{array}$$

where ξ=z/L, defined as the ratio of the reference height *z* and the Obukhov length scale *L*, $a_{h}=b_{h}=5$, $c_{h}=3$, and $B_{h}=\sqrt{5}$. The improved NPS model with the Grachev stability function has produced the best agreement with propagation measurements [45].

The time series at the bottom of Figure 2 is the EDH calculated based on the NPS model, and there is a sudden increase during some stable situations. Zhao et al. [3] found that the NPS model may have errors in calculating EDH when employing the model to assess propagation conditions in strongly stable atmospheric conditions with low wind speeds based on the observation data of the air–sea flux tower on Yongxing Island. Moreover, the observation of the four buoy stations used in this paper shows that the probability of the atmosphere being in unstable atmospheric conditions is basically over 70%, so the EDH statistics are meaningful.

### 2.3. Evaporation Duct Height Entropy

Previously, statistical methods were employed to analyze the probability distribution of EDH differences between stations [37]; while such methods can reveal the likelihood of EDH disparities within a specific range (e.g., −2 to 2 m), they are inherently limited to pairwise comparisons and cannot provide a comprehensive metric for the overall inhomogeneity across multiple stations or a continuous region. Moreover, they do not account for varying degrees of deviation in a unified framework, making it difficult to holistically assess spatial inhomogeneity. To overcome these limitations, we introduce the concept of EDHE based on information entropy. EDHE integrates the deviations of EDH at each grid point from a reference point and weights them by their probability, thereby offering a single scalar that captures the spatial inhomogeneity in a holistic manner. A higher EDHE indicates greater consistency with the reference, while a lower EDHE signifies stronger heterogeneity.

Information entropy is a metric utilized to quantify the unpredictability of phenomena, illustrating the average uncertainty and level of disorder in information sources. It was introduced by Shannon [46], and serves as a key tool in various domains. In meteorology, information entropy finds applications in analyzing spatiotemporal patterns like precipitation [47,48,49] and temperature [50,51,52] distributions. Primarily employed on a temporal scale, it gauges the extent of fluctuations in meteorological factors over time. Essentially, higher information entropy signifies greater variability in the element during the specified timeframe.

This paper refers to the definition of information entropy to introduce the concept of EDHE at a spatial scale. At a certain moment (*t*), the EDHE ($H_{k}$) of a grid point (*k*) is defined as the overall uncertainty of the deviation of the EDH of that grid point from the EDH of the reference grid point at the selected spatial scale. It is expressed as the ratio of the cumulative uncertainty of all moments (*N*) to the total time during a specific time period. The calculation formula is provided as follows:

(2) $$H_{k}=-\frac{\sum_{t=1}^{N}P\left(x_{kt}\right)\log_{10}P\left(x_{\mathrm{kt}}\right)}{N}0<P\left(x_{kt}\right)\leq 1$$

**Figure 3:**
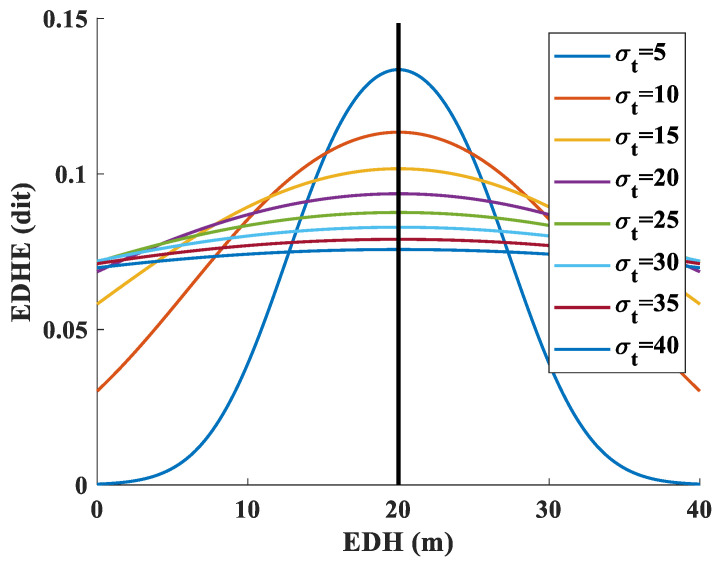
Trend of EDHE variation with the EDH when the EDH of the reference point is 20 m ($x_{t}=20$).

If $P(x_{kt})$ follows a Gaussian distribution, then

(3) $$P\left(x_{kt}\right)=\frac{1}{\sqrt{2\pi \sigma_{t}}}\times e^{-\frac{{\left(x_{kt}-x_{t}\right)}^{2}}{2\sigma_{t}^{2}}}0\leq x_{kt}\leq 40$$

where $H_{k}$ (unit: dit) represents the average EDHE of grid point *k* (*k* = 1, 2, …) over the time period *N*. $x_{kt}$ represents the EDH value of grid point *k* at time *t*, and $P(x_{kt})$ represents the corresponding probability ($0<P(x_{kt})\leq 1$). In the EDH field at time *t*, $\sigma_{t}$ ($\sigma_{t}>0$) represents the standard deviation of EDH at the selected spatial scale and $x_{t}$ represents the EDH of the reference grid point. When the EDH of the reference point is 20 m ($x_{t}=20$), Figure 3 illustrates the trend of EDHE variation with the EDH of other grid points. Different curves in the figure represent the EDHE obtained under varying $\sigma_{t}$ environments. It is evident that the smaller the difference in EDH between the other grid points and the reference point, the larger the EDHE. Therefore, Formula (2) can be interpreted on a spatial scale as follows: when the disparity between the EDH of a specific grid point at a given time and the EDH of the reference grid point is smaller, the information entropy is high. Conversely, greater disparity leads to reduced information entropy.

## 3. Results and Analysis

Evaporation duct models usually work well in unstable conditions, i.e., when the ASTD is negative (ASTD < 0), compared to stable conditions (ASTD > 0). This behavior stems from the fact that evaporation duct models are based on open ocean measurements where unstable conditions are most common [40]. The calculations of EDHs and refractive index profiles are therefore more reliable in unstable conditions. So, the climatology of ASTD for the months and days are prioritized, which may be useful for highlighting areas and times where bulk models are expected to give a reliable EDH. Then, the inhomogeneous characteristics of evaporation ducts in the northern SCS are analyzed.

### 3.1. The Time Distribution Characteristics of ASTD

**Figure 4:**
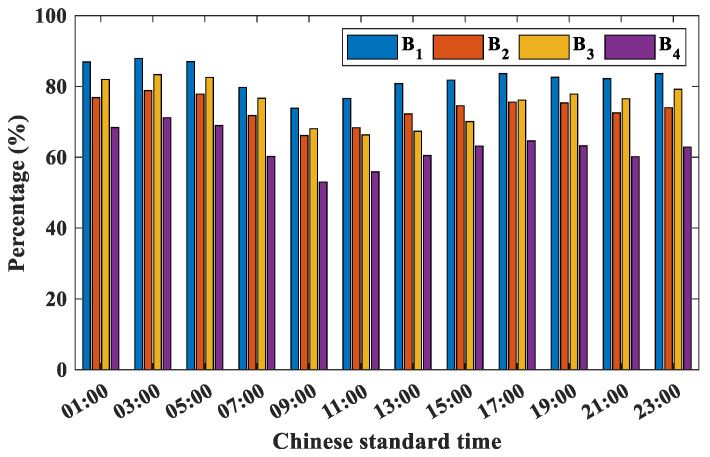
The daily distribution characteristics of ASTD < 0 at buoy stations.

**Figure 5:**
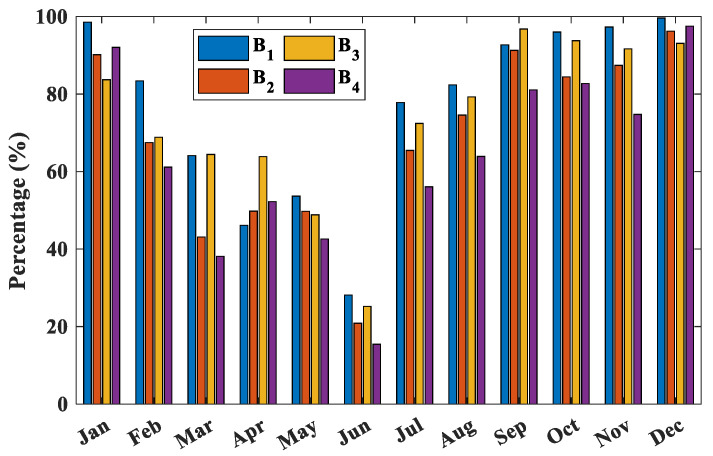
Monthly distribution characteristics of ASTD < 0 at buoy stations.

In this paper, the probability distributions of ASTD < 0 for four buoys are analyzed. Figure 4 and Figure 5 display the daily and monthly probability distributions of ASTD < 0, respectively.

In the daily distribution, station B_1_ consistently shows the highest probability, exceeding 75%, while B_4_ shows lower probabilities of ASTD < 0, but still above 55%. Overall, the percentage of ASTD < 0 decreases for the four buoy stations from 05:00 to 09:00 and then gradually increases. At 09:00, the probability of ASTD < 0 is the lowest for the four buoy stations, with higher percentages during the night. This is primarily caused by the rapid warming of the sea surface (seawater and air) following sunrise, as it absorbs solar shortwave radiation. Due to the high specific heat capacity of seawater compared to the lower specific heat capacity of air, the AT increases more quickly than the temperature of the seawater. This can lead to the formation of a neutral or stable atmospheric state. Conversely, during the night when solar shortwave radiation is absent, the SST continues to decline, while the AT drops faster, causing an unstable atmospheric state. Furthermore, solar shortwave radiation and sea surface WS are identified as the primary factors influencing the seasonal variation in sea surface warming.

For the monthly distribution of ASTD < 0, the probabilities of B_1_ and B_3_ are relatively high. Specifically, the likelihood of B_1_ remaining above 95% from September to January of the following year is notable, while the probability of B_4_ is the lowest. It is worth noting that the probability of B_1_ in January and December exceeds 95%. Conversely, the probability of ASTD < 0 from March to June is relatively low, consistently below 60%. Notably, June sees the lowest probability, dipping below 30%, with ASTD > 0 being more common in the summer and autumn months. However, from July to January of the following year, the probability of ASTD < 0 generally remains above 60%. In the initial months of the year, particularly from March to June, the AT rebounds quicker than the SST, resulting in increased low cloud cover and minimal daily temperature variations. Moreover, the southward winds bring warm and humid air to the northern SCS after the summer monsoon outbreak (May from 2019 to 2022 [53]), enhancing the likelihood of atmospheric stability in the lower atmosphere. Conversely, in the latter half of the year, the AT decreases faster than SST, and the cloud is mainly composed of cumulus clouds with less cloud cover, which increases the probability of ASTD < 0 and makes atmospheric stratification more unstable [54].

In summary, the NPS models may have better accuracy in the evening or autumn/winter seasons in the northern SCS.

### 3.2. The EDH Differences Between Buoy Stations

To facilitate the EDH difference between buoy stations, we designate B_12_ as the EDH difference between B_1_ and B_2_ (B_12_ = B_1_ − B_2_), and B_13_ as the EDH difference between B_1_ and B_3_ (B_13_ = B_1_ − B_3_), respectively. Other definitions are the similar, such as B_14_, B_23_, B_24_, and B_34_.

**Figure 6:**
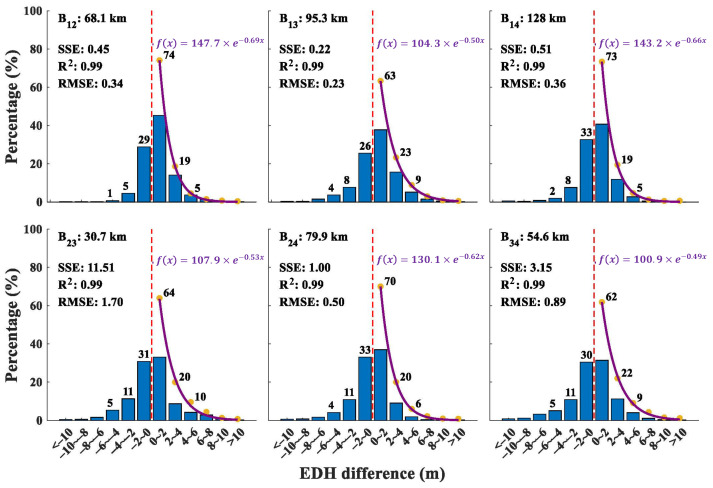
Probability distribution of EDH difference among buoy stations, where the upper left corner of each subgraph represents the corresponding two stations (B_12_ represents stations B_1_ and B_2_; other definitions are the same) and their distances, and the red line in the middle represents the dividing line of EDH difference (left is less than 0; right is greater than 0). The purple line is the probability density function (upper right corner) of the absolute value of the fitted EDH difference; the SSE, R^2^, and RMSE are the error sum of squares (SSE), R-Square (R^2^), and Root Mean Square Error (RMSE) of the probability density function, respectively. The yellow dots represent the percentage of the absolute value of the EDH differences between different stations across different intervals.

A histogram of the EDH difference between buoy stations is depicted in Figure 6. It is worth noting that the probability of EDH differences ranging from −2 m to 2 m is over 70% for B_12_, B_14_, and B_24_, with corresponding distances of 68.1 km, 128 km, and 79.9 km. The lowest probability is observed for B_34_, with a probability and distance of 62% and 54.6 km, respectively. On the whole, the average probability of EDH differences between −2 m to 2 m is 67.7%, and the probability that the EDH difference is positive is above 50%, which indicates that the EDH values tend to be slightly higher in the northern regions of the study area in most instances.

Moreover, a fitting of the probability distribution of the absolute value of EDH differences between buoy stations (shown by the purple line in Figure 6) was conducted, and the error sum of squares (SSE), R-Square (R^2^), and Root Mean Square Error (RMSE) of the probability density function were computed. In general, the probability distribution of the absolute value of EDH differences between buoy stations conforms to an exponential distribution form $f\left(x\right)=A\times e^{-Bx}$, where *x* denotes the absolute value of EDH difference between buoy stations, and *A* and *B* are the fitting parameters. Analysis of the six exponential distribution functions reveals that *A* is typically greater than 100, while *B* is generally less than 0.7. Furthermore, the average SSE, R^2^, and RMSE resulting from the fitting process are 2.8, 0.99, and 0.67, respectively.

**Figure 7:**
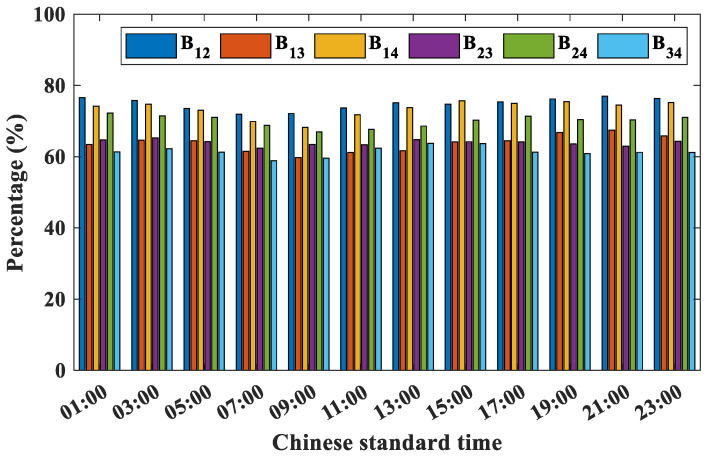
Daily distribution characteristics of EDH differences between −2 m to 2 m among buoy stations.

This study conducts statistical analysis to analyze the time distribution characteristics of the EDH difference falling within the range of −2 m to 2 m among the four buoy stations. Figure 7 illustrates that the probability of the EDH difference falling within −2 m to 2 m among the four buoy stations remains relatively constant, such as B_12_ maintaining approximately 78%. This suggests that the horizontal inhomogeneity of the evaporation duct does not exhibit significant variations over time. Furthermore, due to the low proportion of ASTD < 0 from March to June, no monthly distribution statistics were conducted for this period.

Due to the likelihood of observing ASTD < 0 exceeding 60% from the data collected by the four buoy stations, and the superior performance of the evaporation duct model in unstable atmospheres, a Pearson correlation analysis of the key factors contributing to the inhomogeneous distribution of the evaporation duct when ASTD < 0 was carried out. Table 4 displays the Pearson correlation coefficients between the difference in EDH among buoy stations and RH, WS, SST, and AT. The disparities in EDH among buoy stations are predominantly influenced by WS and RH, with respective average Pearson correlation coefficients of 0.69 and −0.63, while the influence of SST and AT on EDH variances is minimal. Furthermore, the Pearson correlation coefficients between the difference in EDH at B_2_ and other stations and RH are notably high, indicating a significant divergence in RH at B_2_ compared to the other stations, possibly attributable to rainfall. In conclusion, the principal meteorological factor contributing to the inhomogeneous EDH in the research area is WS, followed by RH.

The strong correlation between EDH differences and wind speed (Table 4) suggests that the spatial inhomogeneity of EDH in the study area may largely reflect the inhomogeneity of the near-surface wind field. Further investigation into the mechanisms driving wind field variability—such as land–sea breeze circulations or synoptic weather systems—would be valuable for understanding the origins of EDH spatial patterns, and represents a promising direction for future research using high-resolution atmospheric models or dedicated field observations.

### 3.3. The Evaporation Duct Height Entropy in the Northern South China Sea Based on Buoy Stations

**Figure 8:**
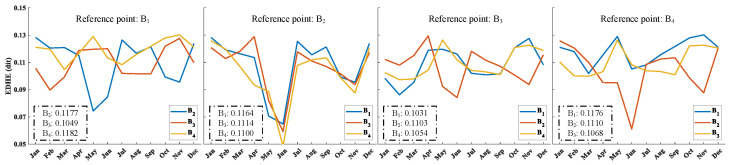
Monthly distribution of EDHE between buoy stations at different reference points; the content within the dashed line represents the average EDHE.

Based on statistical methods, we analyzed the probability distribution of EDH differences between stations (Figure 6). However, it remains unclear which two stations had the smallest EDH differences. To clarify, we calculated the EDHE between the reference station and other stations, using stations B_1_, B_2_, B_3_ and B_4_ as reference stations, respectively. The monthly distribution of EDHE with these reference stations is illustrated in Figure 8. When station B_1_ is used as a reference station, the average EDHE values for stations B_2_, B_3_, and B_4_ are 0.1177, 0.1049, and 0.1182, respectively. The monthly distribution of EDHE between stations B_1_ and B_4_ is relatively stable, while the EDHE between stations B_1_ and B_2_ is notably lower in May and June. When station B_2_ serves as the reference point, the EDHE values for B_2_ and the other stations are also relatively low in May and June, indicating a significant difference in EDH between station B_2_ and the others during that time. With station B_3_ as the reference point, the average EDHE between stations B_3_ and B_4_ is the highest, whereas the EDHE between stations B3 and B_2_ is the lowest. Similarly, when using station B_4_ as the reference point, the average EDHE between station B_4_ and station B_1_ is the highest, while the EDHE between station B_4_ and station B_2_ is the lowest. In summary, the difference in EDH between station B_1_ and station B_4_ is minimal.

By comparing Figure 6 and Figure 8, it is evident that the probability distribution of the EDH difference between the reference station and other stations, within the range of −2 to 2 m, closely aligns with the corresponding rankings of the average EDHE. For instance, the probability of the EDH difference between station B_1_ and the other stations within this range is 74% (B_12_), 63% (B_13_), and 73% (B_14_), respectively. In contrast, the corresponding average EDHE values are 0.1177, 0.1049, and 0.1182. This indicates that higher probabilities generally correspond to higher EDHE values; however, the EDHE metric provides a better measure of inhomogeneity between stations. In summary, EDHE serves as an effective indicator of the differences in EDH between stations, further emphasizing the inhomogeneous characteristics of EDH.

### 3.4. The Evaporation Duct Height Entropy in the Northern South China Sea Based on Reanalysis Data

The inhomogeneity characteristics of EDH across different stations can be assessed using buoy station data; however, analyzing the regional inhomogeneity of EDH necessitates the use of reanalysis data. This section utilizes reanalysis data to analyze the inhomogeneity characteristics of EDH in the northern SCS region. It should be pointed out that the EDHE is calculated based on the grid point where station B_4_ is located as the reference station in this section.

#### 3.4.1. Validation Analysis of Reanalysis Data

**Figure 9:**
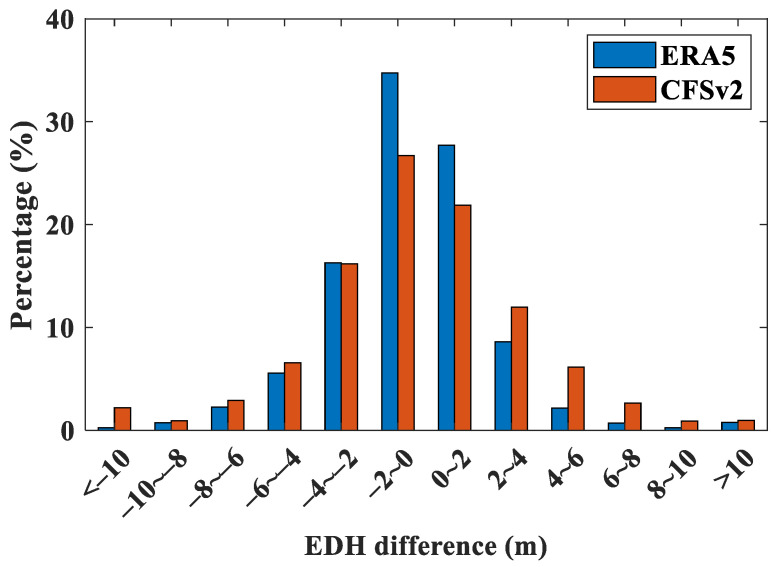
Probability distribution of differences between EDH obtained from reanalysis data and buoy stations.

The buoy is a prevalent device used for oceanic atmospheric detection, but setting up buoy observation points on a large scale is challenging due to the unique nature of the ocean. As a result, research and analysis of the evaporation duct mechanism primarily rely on reanalysis data. The commonly utilized reanalysis datasets at present are CFSv2 and ERA5. However, the suitability of these datasets in the northern SCS region needs further validation. Therefore, this section conducted a comparative analysis of the applicability of these two datasets based on buoy observations. The EDH is influenced by AT, RH, SST, and WS. Because of the complex interactions among these meteorological factors, determining which dataset provides the most accurate calculation of the EDH based solely on the consistency of individual meteorological elements is challenging. Therefore, the EDHs for both buoy and reanalysis datasets based on the NPS model are calculated. Then, the EDHs obtained from the reanalysis dataset with the buoy data are compared, and the differences in EDHs are statistically analyzed (Figure 9). Figure 9 demonstrates that the EDH calculated from the ERA5 dataset closely aligns with the buoy data, with a probability of the EDH difference falling between −2 m and 2 m exceeding 60%. In contrast, the EDH derived from the CFSv2 dataset shows relatively higher errors. Furthermore, the probability of EDH differences being less than 0 consistently exceeds 50%, indicating that in most cases, reanalysis data tend to overestimate the EDH. In conclusion, the ERA5 dataset shows better consistency with the buoy observations of the evaporation duct in the study area. Therefore, this dataset is used to analyze the inhomogeneous characteristics of the evaporation duct in the northern SCS.

#### 3.4.2. The Monthly Distribution Characteristics of EDH

**Figure 10:**
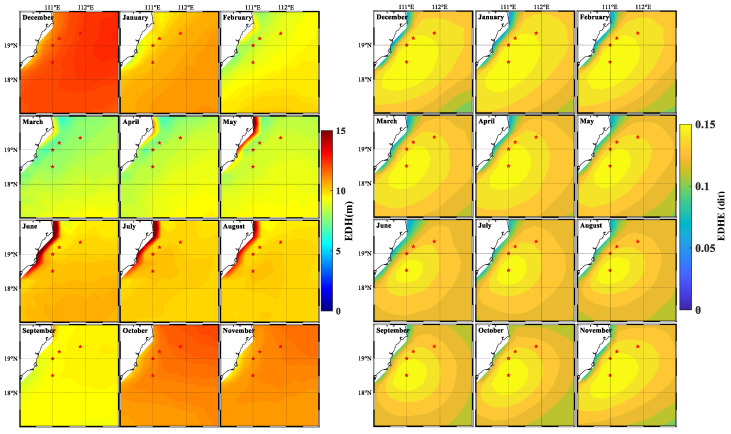
Monthly distribution characteristics of EDH (**left**) and EDHE (**right**) in the research area. Among them, the red pentagram represents the position of the buoy station.

To facilitate the understanding of the monthly variation characteristics of the EDHE, the monthly average distribution of the EDH and the monthly average distribution of the EDHE from 2014 to 2023 were calculated (Figure 10). The monthly average distribution of EDH (Figure 10 left) displays distinct seasonal variations. During summer and autumn, the EDH is lower compared to spring and winter, with the lowest average height occurring in summer (less than 10 m). Along the coast, the EDH also fluctuates with the seasons. In summer and autumn, the EDH is higher than in other regions, while in spring and winter, the coastal areas’ EDH is lower than in other areas. The sea–land breeze circulation primarily influences the variation in EDH in coastal areas [55].

#### 3.4.3. The Monthly Distribution Characteristics of EDHE

Using the grid point where station B_4_ is located as the reference point (Figure 10 right), it is observed that the high-value area of EDHE is more extensive in spring and relatively smaller in autumn. Additionally, the high-value area of EDHE demonstrates a gradual decrease from January to June, followed by an increase from June to December. Consequently, in June, the horizontal distribution of EDH in the region is the most inhomogeneous, which may be attributed to the onset of the SCS summer monsoon, which typically intensifies in June. The interaction between the summer monsoon and the land–sea dynamics contributes to the inhomogeneity of EDH across the region’s horizontal distribution. Spatially, the distribution of EDHE largely exhibits a long, strip-like pattern extending from northeast to southwest, aligning with the coastal shape in the southeastern area of Hainan Island. This further emphasizes the influence of sea breeze and land breeze interactions on EDH in the coastal zone.

#### 3.4.4. Comparison of Monthly Distribution of EDHE Based on Different Datasets

**Figure 11:**
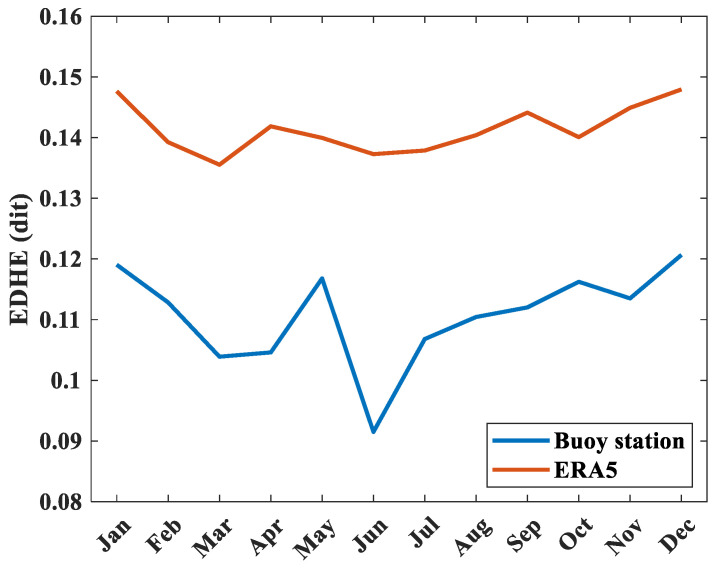
Monthly distribution characteristics of EDHE. It should be pointed out that the EDHE is calculated based on the grid point where station B_4_ is located as the reference station.

To validate the accuracy of the monthly variation in EDHE based on ERA5 data, the EDHEs of the buoy stations and ERA5 were compared, as depicted in Figure 11. To obtain AT, RH, AP, and SST at the buoy stations from ERA5, we employed bilinear interpolation and a distance weighting method to calculate these meteorological elements with an hour temporal resolution. The analysis reveals a strong correlation in the monthly fluctuations between the two datasets; however, ERA5 tends to overestimate the EDHE.

## 4. Discussions

An evaporation duct is a common abnormal propagation condition at sea; the average probability in the SCS is above 85% [2,23], and the occurrence at night is higher than that in the daytime. Moreover, the microwave link over the ocean was constructed in northern SCS during a specific period using an evaporation duct to realize wireless communication, and the efficient connection rate reached 90% [9,23], with the occurrence probability of the evaporation duct being about 90% based on the iron tower platform [56,57]. Evaporation ducts are permanent in offshore winds under unstable conditions, with a percentage of occurrence up to 100%, and their occurrence percentage is above 80% under offshore winds combined with stable conditions [55]. Furthermore, the NPS model is widely applied for its superior accuracy in evaporation duct and electromagnetic propagation research [6,30,53,58]. In the observation of buoy stations, the proportions of ASTD < 0 corresponding to B_1_, B_2_, B_3_ and B_4_ are 83.3%, 75.5%, 73.6%, and 62.7%, respectively. Therefore, the statistical analysis of the EDH differences between buoy stations is meaningful.

When determining the research area and reference point, the closer the grid point EDH is to the reference point, the larger its EDHE. It is crucial to account for the impact of their inhomogeneity on communication quality when considering the use of evaporation ducts for offshore microwave communication. For instance, before establishing a maritime communication network based on buoy stations, the inhomogeneity characteristics of the EDH in the proposed communication area can be assessed using EDHE derived from ERA5 data to determine the optimal network layout. Furthermore, in practical communication scenarios, the inhomogeneity of EDH significantly impacts the quality of beyond-line-of-sight communication. This effect can also be quantitatively analyzed using EDHE. Unfortunately, the lack of electromagnetic propagation data between buoy stations has hindered the ability to conduct thorough research on electromagnetic propagation in inhomogeneous environments. Moving forward, there will be a focus on establishing a communication network among sea buoys and delving deeper into the study of electromagnetic propagation characteristics in evaporation duct environments.

It should be noted that the specific spatial patterns (e.g., the June minimum inhomogeneity and the northeast–southwest strip-like distribution) identified in this study are based on a limited area east of Hainan Island and may not be directly extrapolated to the entire northern South China Sea or other ocean basins. However, the EDHE methodology itself is universally applicable and can be readily extended to larger regions using reanalysis or satellite data in future investigations.

## 5. Conclusions

In this paper, the daily and monthly distribution patterns of ASTD < 0 at four buoy stations located in the northern SCS were examined, analyzing distinctive temporal distribution patterns for ASTD < 0 and variations in EDH among these buoy stations based on the NPS model. The results show that ASTD < 0 occurrences were more prevalent during nighttime and in the spring and winter seasons, and the probability of EDH differences between buoys falling within the range of −2 m to 2 m exceeded 60%, with minimal fluctuations throughout the day. Furthermore, the study compared the accuracy of ERA5 and CFSv2 data in calculating EDH, highlighting the superior precision of ERA5 data in calculating EDH, with probabilities of EDH differences between buoys and ERA5 falling within the range of −2 m to 2 m consistently exceeding 60%. Additionally, the concept of information entropy was introduced to characterize the inhomogeneity of EDH, leading to the definition of EDHE. The efficacy of EDHE in capturing EDH inhomogeneity was validated using buoy data. An examination of the EDH inhomogeneity characteristics in the northern SCS from 2014 to 2023 based on ERA5 data revealed significant monthly distribution features. The analysis indicated a more uniform distribution of evaporation ducts in the northern SCS areas during the spring and winter.

For this study, the NPS model has limitations in stable environments constrained by the use of MOST. In addition, the temporal analysis focused on seasonal and diurnal variations, while interannual variability was not examined due to the limited duration of the buoy observations. Hence, the results may not be directly generalizable to other times or regions. Future efforts should aim to substantiate the universality of these findings by employing longer time series from reanalysis data or sustained observational networks and by improving model performance under stable conditions.

## Figures and Tables

**Table 1:** Sensor specifications (the SST sensor is installed at 0 m height because it is a conductivity, temperature, and depth (CTD) sensor).

Parameter	Sensor	Precision of Buoy Stations	Installation Height
Air Temperature (AT)	Vaisala HMP155	±0.3 °C	5 m
Relative Humidity (RH)	Vaisala HMP155	±2%	5 m
Wind Speed (WS)	Young 05106	±0.3 m/s	6 m
Air Pressure (AP)	Vaisala PTB210	±0.3 hPa	2.9 m
Sea Surface Temperature (SST)	JFE Compact-CTD Lite	±0.01 °C	0 m

**Table 2:** Mean value and standard deviation of the buoy station (B_1_).

Parameter (Unit)	Mean	Standard Deviation
SST (°C)	27.2	2.6
AT (°C)	26.3	3.0
ASTD (°C)	−0.9	1.0
RH (%)	81.5	6.5
AP (hPa)	1011.3	5.7
WS (m/s)	6.9	3.0
EDH (m)	10.0	3.2

**Table 3:** ERA5 and CFSv2 data information used in this paper.

Item	ERA5	CFSv2
Temporal Span	2014∼2023	2019∼2022
Horizontal Resolution	0.25° × 0.25°	0.2° × 0.2°
Meteorological Elements	10 m u/v component wind, 2 m air temperature, 2 m dewpoint temperature, sea surface temperature, surface pressure, 2 m specific humidity.	
Data Type	Conventional longitude and latitude grid data	
Time Resolution	1 h	
Spatial Range	110° E∼113° E, 17° N∼20° N	

**Table 4:** Pearson correlation coefficients between the difference in EDH among buoy stations and RH, WS, SST, and AT when ASTD < 0.

Buoy Stations	RH	WS	SST	AT
B_12_	−0.73	0.54	0.12	0.22
B_13_	−0.54	0.78	0.27	0.30
B_14_	−0.51	0.75	0.12	0.14
B_23_	−0.67	0.70	0.52	0.51
B_24_	−0.73	0.54	0.06	0.15
B_34_	−0.59	0.82	0.59	0.59
Mean	−0.63	0.69	0.28	0.32

## Data Availability

The original contributions presented in this study are included in the article. Further inquiries can be directed to the corresponding author.
